# Special Issue “Managing Dry Eye Disease over Time: An Italian Consensus Conference”

**DOI:** 10.3390/jcm11092507

**Published:** 2022-04-29

**Authors:** Pasquale Aragona, Giuseppe Giannaccare, Maurizio Rolando

**Affiliations:** 1 Department of Biomedical and Dental Sciences and Morphofunctional Imaging, Regional Referral Center for the Ocular Surface Diseases, University of Messina, 98121 Messina, Italy; pasquale.aragona@unime.it; 2 Department of Ophthalmology, University Magna Graecia of Catanzaro, 88100 Catanzaro, Italy; 3 Ocular Surface Centre, ISPRE Ophthalmics, 16129 Genoa, Italy; maurizio.rolando@gmail.com

Dry eye disease (DED) is a chronic, progressive, highly prevalent condition affecting 5 to 33% of the global adult population [1]. After the first comprehensive definition was published in 1995 on the basis of consensus from the National Eye Institute [2], it was realized that the complexity of DED tended to be underestimated and therefore the disease underdiagnosed. Two major steps forward were taken in 2007 [3] and 2017 [4], and the results were published in the reports of DEWS and DEWS II of the TFOS, respectively. Nowadays, it is clear that DED affects not only tears, but the entire ocular surface system, and that three main pathogenic factors, namely tear instability, inflammation and damage to the epithelia, play a key role for its development; these factors can be associated, as a cause or as a consequence, with eyelid abnormalities and nerve dysfunction [5].

Another important aspect of DED is related to the symptoms referred by the patients that can significantly impair patients’ quality of life and daily activities [6,7]. Furthermore, after the first diagnosis and the initial therapeutic program, patients feel alone in the face of a disease that will have ups and downs over time with sudden flare-ups. To improve patients’ satisfaction, it is essential not only to prescribe the proper therapy but also to monitor the course of signs and symptoms over time in order to adapt the treatment according to the response with the aim of avoiding the chronicization of the disease [8].

Counseling, patient education and doctor–patient alliance are important tools that can promote therapeutic efficacy, which is crucial to guarantee adherence to therapy and a reduction in symptoms [9].

In the Italian context, clinicians felt the need to define and share a standard approach to the diagnosis, prevention and treatment of DED. In order to address this unmet need, a group of ophthalmologists who contend with ocular surface disease issues on a daily basis (“Italian Dry Eye Consensus Group”) has worked together in creating a new project named “Eye Care 4 Care”. This expert group convened to a consensus on practical algorithms for providing treatment recommendations, reached by means of a Delphi consensus process, in four different scenarios: (i) “DED and inflammation”; (ii) “DED and eyelid”; (iii) “DED and surgery”; (iv) “DED and treatment compliance”. Despite every scenario being different, creating some algorithms will assist in coming to the right answer over time. In order to respect the complexity and the numerous settings in which the condition of DED can be found, the scientific data obtained by evidence available in the literature and also the clinical experience of “real life” have been considered through an extended and shared discussion. Monitoring the status of the patient over time can show if there is a trend of improvement or worsening, and if the target is reached or not. This will allow tailoring the treatment according to the different clinical conditions presented by a single patient at each time point. It is intended that these algorithms will be useful in a clinical setting for general ophthalmologists.

There are still many fundamental questions that need to be addressed in order to improve DED treatment effectiveness over time and therefore patients’ satisfaction. This Special Issue will describe in detail all the four algorithms proposed by the Italian Dry Eye Consensus Group along with other open issues related to the management over time of DED.

As Guest Editors, we would like to give special thanks to the colleagues that took part in this project for their valuable contributions and to the *Journal of Clinical Medicine* team for their robust support.
